# Diagnostic Accuracy of Computer-Aided Detection of Pulmonary Tuberculosis in Chest Radiographs: A Validation Study from Sub-Saharan Africa

**DOI:** 10.1371/journal.pone.0106381

**Published:** 2014-09-05

**Authors:** Marianne Breuninger, Bram van Ginneken, Rick H. H. M. Philipsen, Francis Mhimbira, Jerry J. Hella, Fred Lwilla, Jan van den Hombergh, Amanda Ross, Levan Jugheli, Dirk Wagner, Klaus Reither

**Affiliations:** 1 Swiss Tropical and Public Health Institute, Basel, Switzerland; 2 Ifakara Health Institute, Bagamoyo, United Republic of Tanzania; 3 Center for Infectious Diseases and Travel Medicine, University Hospital Freiburg, Freiburg, Germany; 4 Diagnostic Image Analysis Group, Radboud University Medical Center, Nijmegen, The Netherlands; 5 PharmAccess International, Dar es Salaam, United Republic of Tanzania; 6 University of Basel, Basel, Switzerland; National Institute of Infectious Diseases, Japan

## Abstract

**Background:**

Chest radiography to diagnose and screen for pulmonary tuberculosis has limitations, especially due to inter-reader variability. Automating the interpretation has the potential to overcome this drawback and to deliver objective and reproducible results. The CAD4TB software is a computer-aided detection system that has shown promising preliminary findings. Evaluation studies in different settings are needed to assess diagnostic accuracy and practicability of use.

**Methods:**

CAD4TB was evaluated on chest radiographs of patients with symptoms suggestive of pulmonary tuberculosis enrolled in two cohort studies in Tanzania. All patients were characterized by sputum smear microscopy and culture including subsequent antigen or molecular confirmation of *Mycobacterium tuberculosis* (M.tb) to determine the reference standard. Chest radiographs were read by the software and two human readers, one expert reader and one clinical officer. The sensitivity and specificity of CAD4TB was depicted using receiver operating characteristic (ROC) curves, the area under the curve calculated and the performance of the software compared to the results of human readers.

**Results:**

Of 861 study participants, 194 (23%) were culture-positive for M.tb. The area under the ROC curve of CAD4TB for the detection of culture-positive pulmonary tuberculosis was 0.84 (95% CI 0.80–0.88). CAD4TB was significantly more accurate for the discrimination of smear-positive cases against non TB patients than for smear-negative cases (p-value<0.01). It differentiated better between TB cases and non TB patients among HIV-negative compared to HIV-positive individuals (p<0.01). CAD4TB significantly outperformed the clinical officer, but did not reach the accuracy of the expert reader (p = 0.02), for a tuberculosis specific reading threshold.

**Conclusion:**

CAD4TB accurately distinguished between the chest radiographs of culture-positive TB cases and controls. Further studies on cost-effectiveness, operational and ethical aspects should determine its place in diagnostic and screening algorithms.

## Introduction

The role and potential of chest radiography as a diagnostic and screening tool for pulmonary tuberculosis (PTB) has long been debated. As a rapid examination that can be interpreted on-site with a high sensitivity (between 74 and 90% for PTB related abnormalities, up to 97%, if any abnormality is considered [1]–[4]), it has a firm place in the diagnosis of pulmonary tuberculosis. However, the lower specificity, a lack of consistency in how results are reported and high levels of inter- and intra-reader variability are matters of concern. Interpreting chest radiographs is complex and subjective: it is a two dimensional representation of a three dimensional structure, and there are varied manifestations of PTB. The complexity of the interpretation code and the structure of the report form affect the result [5], [6]. Different readers are also influenced by experience and professional training [7], [8] and momentary factors like distraction, focus and tiredness.

In contrast, the automated reading of radiographs by computers is devoid of inter- and intra-observer variability. Research in this field started fifty years ago. Although early optimistic goals such as “fully automating the chest exam” [9] are still far from being achieved, at least one application, the automatic detection of masses and micro-calcifications in mammograms, has been successfully integrated in clinical routine to support radiologists in their decision [10].

Most of the research on computer-aided diagnosis (CAD) of chest radiographs focuses on the detection of nodules, but there are a number of research groups also working on promoting CAD in PTB. Among these, the Diagnostic Image Analysis Group at Radboud University Medical Center, Nijmegen, The Netherlands introduced CAD4TB, a software to determine whether a chest X-ray (CXR) shows evidence of PTB. CAD4TB underwent field tests in 2010 and has been developed since then. Previous software versions were comparable to clinical officers for detecting culture confirmed tuberculosis (TB) among 166 presumptive TB patients at a Zambian clinic (v1.08; area (A_z_) under the receiver operating characteristic (ROC) curve = 0.73) [11] and reached a sensitivity of 95% at a specificity of 57% in 95 CXRs of homeless people in London (texture abnormality detection system; A_z_ = 0.86) [12].

A recently published review article on automatic screening for tuberculosis in chest radiographs by Jaeger and colleagues [13] concludes that even though proposed CAD algorithms seem to perform reasonably well when tested individually, no fair comparison can be made without testing the systems on the same, preferably large and publicly available dataset of well characterized patients. The authors further emphasise that there are hardly any validation studies from clinical or screening situations so far and therefore a lack of evidence on how the systems perform in the practical field.

We conducted the first validation study to assess the diagnostic accuracy of the most recent CAD4TB software (v3.07, updated release) on a large set of well characterized adult presumptive PTB patients from sub-Saharan Africa. We compared the performance of the automated reading with the results of human observers of different experience levels.

## Methods

### Study Population

This validation study was done on chest radiographs of participants from two cohort studies (TB Cohort and TB CHILD study) which have been conducted at the TB Clinic of the Ifakara Health Institute (IHI) in Bagamoyo, Tanzania. Tanzania has a high burden of active TB: according to the first national Tuberculosis Prevalence Survey in 2013 the prevalence is 295 cases per 100,000 population [14]. Bagamoyo, a town of 35,000 inhabitants, is located on the coast, approximately 70 km from the commercial capital Dar es Salaam.

Individuals presenting with clinical signs and symptoms suggestive of pulmonary TB to surrounding primary health care facilities were referred to the IHI TB Clinic. Patients who met the inclusion criteria and gave informed consent were consecutively enrolled into either the TB Cohort or TB CHILD study. In both studies the patients were followed up for 5 to 18 months. The main objective of the TB Cohort study was to generate a sound understanding of TB epidemiology in the Bagamoyo region, while the TB CHILD study was conducted to assess performance characteristics of new TB diagnostics in adults and children. Written informed consent was obtained from all literate patients. In case of illiteracy, informed oral consent was attested by an impartial witness and documented with the patient's fingerprint according to ICH GCP guidelines as approved by the IHI Institutional Review Board and the Medical Research Coordinating Committee of the National Institute for Medical Research, Tanzania. Patients who received anti-TB treatment during the last year, were severely sick or did not reside within the study area were not included. All adult patients from both studies were eligible for the CAD4TB validation study if they initially presented with persistent cough of 2 weeks or more and at least one of the following TB associated findings: haemoptysis, chest pain, fever, night sweats, constant fatigue, recent unexplained weight loss, loss of appetite, malaise or contact with a known TB case.

### Specimen collection & Laboratory methods

At enrolment, the participants answered a detailed questionnaire about their medical history, underwent a clinical examination, had a chest radiograph taken and sputum and blood samples were collected. All CXRs (resolution: 1760×2140 pixel) were taken with a Philips Cosmos BS radiography system, which operated combined with a Philips PCR System Eleva S processor. Two sputum specimens, one ‘spot’ and one early morning, were routinely obtained and used for acid-fast bacilli (AFB) smear and culture examination. All samples were decontaminated using the standard NALC-NaOH method, inoculated on both solid (Löwenstein-Jensen, LJ) and liquid (Mycobacterium Growth Identification Tube, MGIT) media and incubated at 37°C. Smears were performed from the decontaminated pellet, followed with Ziehl-Neelsen (ZN) staining. All positive cultures were tested by ZN microscopy for the presence of AFB, and *Mycobacterium tuberculosis* (M.tb) was confirmed by MPT64 antigen and/or molecular tests (Genotype MTBC, CM or AS; Hain Lifescience, Nehren). Interpretation of all microbiological tests was carried out blind to clinical information and radiological results. Voluntary HIV counselling and testing was offered to all participants. The laboratory work was carried out according to Good Clinical Laboratory Practice to guarantee objective standards, quality control and assurance.

### Classification

All patients were classified by the study physicians (M.D., 1–3 years of clinical experience) in consultation with a senior physician (M.D., 20 years of clinical experience) into seven groups (table 1) according to all clinical and microbiological information available 5 months after enrolment. Allocation to the groups was not mutually exclusive. For the purpose of this analysis, it was agreed that classification to either group A (s+/c+ M.tb) or B (s−/c+ M.tb) supersedes classification to C (s ±/c+ NTM) or E (EPTB), and classification to either group G (Indeterminate) or D (s−/c− clin.TB) supersedes classification to group C (s ±/c+ NTM). Patients with resolved symptoms after 5 months and who were confirmed to be definitely free of TB (group F) will be referred to as ‘Controls’ in the following.

**Table 1:** Classification of study population according to clinical and microbiological data.

Group	Description	Short form
A	Smear positive/culture positive, *Mycobacterium tuberculosis*	s+/c+ M.tb
B	Smear negative/culture positive, *Mycobacterium tuberculosis*	s−/c+ M.tb
C	Smear negative or positive/culture positive, nontuberculous mycobacteria (NTM), irrespective of clinical relevance	s ±/c+ NTM
D	All cultures negative, CXR and clinical symptoms very suspect for PTB (clinically diagnosed TB)	s−/c− clin.TB
E	Cytologically/histologically/microbiologically confirmed extrapulmonary TB	EPTB
F	All smears and cultures negative and sustained recovery up to 5 months (e.g. resolved bronchitis or pneumonia)	Controls
G	Loss to follow-up after recruitment or any other combination of results (e.g. still symptomatic after 5 months)	Indeterminate

### Reading of the chest radiographs

The computer-aided analysis of the CXRs was performed independently and blind to clinical information and radiological results by the Diagnostic Image Analysis Group at Radboud University Medical Center, Nijmegen, The Netherlands. The images were processed with the latest CAD4TB software version (v3.07, updated release). CAD4TB is a software framework in which various subsystems for the detection of textural and shape abnormalities, for symmetry and correlation analyses operate at pixel and image level [15].

In CAD, the analysis is broken down to several computable steps [16]: First, radiographs are pre-processed to normalise image features like resolution and grey scale. During segmentation, the next step, the software seeks the anatomical orientation of the image by demarcating structures like the lungs, clavicles and ribs. The defined lung fields are then analysed for their shape, global symmetry and local texture. In addition, a global correlation with a typical normal CXR is determined. Scores generated by these subsystems are combined to an overall score for each image which summarises the result of the automated analysis as an abnormality score for the presence of active disease between 0–100.

In addition, the same set of images was read by two human observers: one experienced chest physician as expert reader and one clinical officer who had practical experience in reading chest X-ray exams in his role as District Tuberculosis and Leprosy Coordinator and had completed a one week course on “X-ray interpretation of tuberculosis and HIV-related opportunistic infections among people living with HIV” [17]. The two readers rated the images using the ‘Tanzanian X-ray score’, a template for a structured CXR report. At the end of their report, the readers were asked to choose between four possible conclusions:

1. normal.
2. abnormal, findings not suggestive for active TB (TB sequel possible).
3. abnormal, findings consistent with active TB, but TB sequel or other lung pathology possible.
4. abnormal, findings highly suggestive for active TB.

Three different reading thresholds were defined correspondingly, ranging from considering only ‘abnormalities highly suggestive for TB’ (conclusion 4) to ‘TB consistent abnormalities’ (conclusion 3+4) to ‘any abnormality’ (conclusion 2–4) for a positive test result.

The readings of chest radiographs were carried out retrospectively for both, the automated and the human interpretation, and had no influence on the diagnosis of the study participants. The human readers were only aware of the inclusion criteria of the study and the age of the patients but blind to clinical information, bacteriological results as well as each other's results.

### Data analysis

Culture-confirmed M.tb was used as a reference standard to assess the diagnostic accuracy of CAD4TB and the human readers for the diagnosis of PTB. Individuals whose state of disease could be definitely determined were included in the analyses: group A (s+/c+ M.tb) and B (s−/c+ M.tb) as true cases and group F (Controls) as definite non TB patients. Secondary performance analyses were carried out in which individuals of group C (s ±/c+ NTM) and E (EPTB) were considered additionally to group F (Controls) to be most likely free of pulmonary TB. Individuals of group D (s−/c− clin.TB) were classified partly due to an abnormal X-ray and were excluded from the analysis.

Receiver operating characteristic (ROC) curves and their areas under the curve (A_z_) were calculated based on the output of the software. Their 95% confidence intervals (CI) and p-values were computed using the De Long method [18]. The performance of the human readers was summarised by calculating sensitivities, specificities, positive and negative predictive values as well as diagnostic likelihood ratios and their 95% confidence intervals for reporting ‘abnormalities highly suggestive for TB’ (conclusion 4), ‘TB consistent abnormalities’ (conclusion 3+4) or ‘any abnormality’ (conclusion 2–4). The same performance measures were calculated for several exemplary cut-offs of the CAD4TB software. Proportions in different groups were compared using the chi-squared test. McNemar's test was applied to compare the specificity of CAD and humans at assumed levels of sensitivity. Mann-Whitney-Wilcoxon test was used to compare the CAD scores between different groups. All calculations were done using the statistical package ‘R’, version 3.0.0 [19] together with the extension packages ‘pROC’ [20], ‘epiR’ [21], ‘ggplot2’ [22], ‘reshape2’ [23] and ‘plotrix’ [24]. All data used for the analyses is deposited in a public repository and can be accessed via http://dx.doi.org/10.6084/m9.figshare.936571.

### Ethical considerations

The TB Cohort and TB CHILD studies were approved by the IHI Institutional Review Board and the Medical Research Coordinating Committee of the National Institute for Medical Research, Tanzania.

## Results

**Figure 1:**
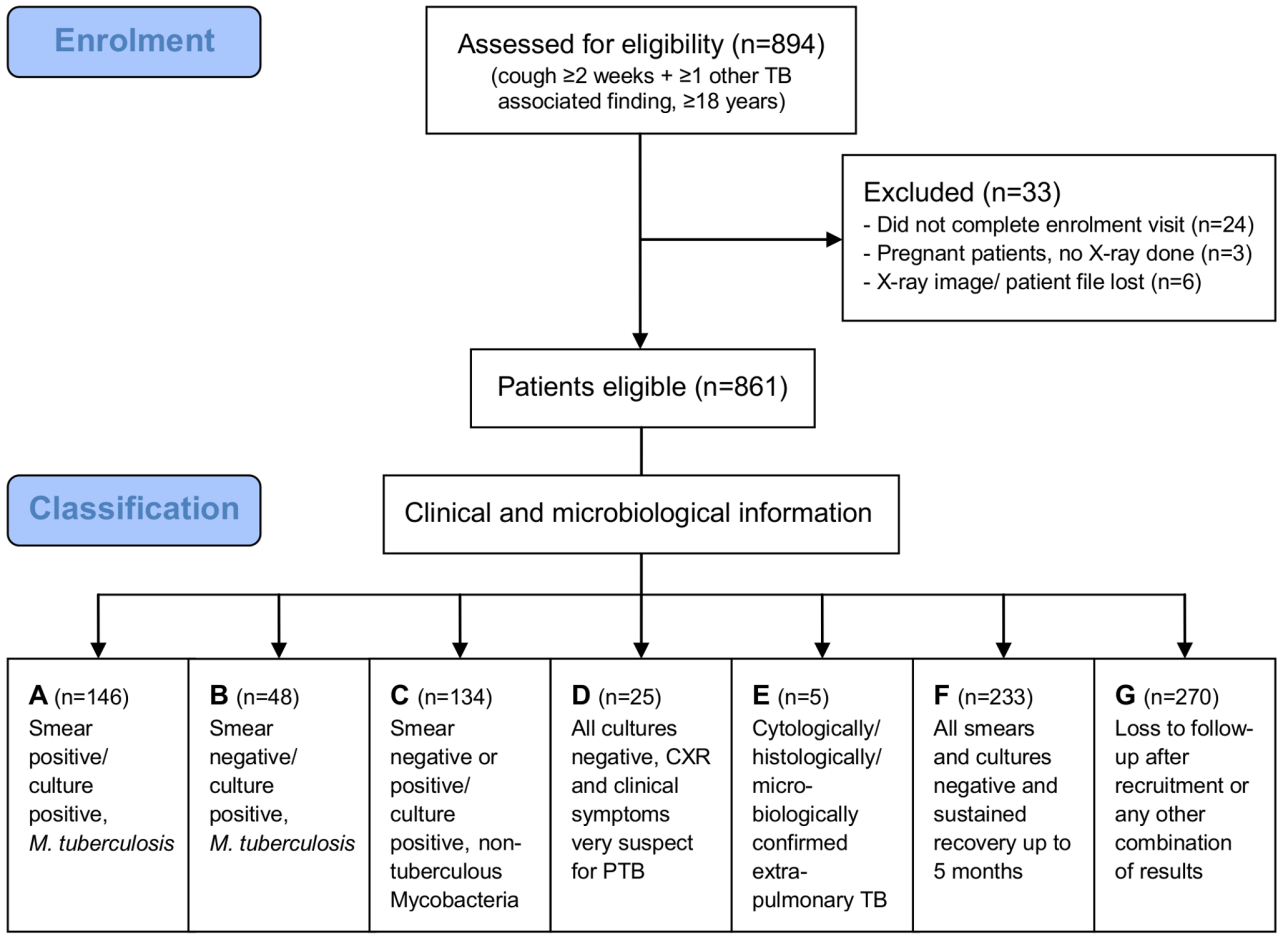
Flow chart of individuals taking part in the study.

A total of 894 patients were enrolled in the CAD4TB validation study. Thirty-three patients had to be excluded from analysis because of an incomplete enrolment visit, pregnancy or missing chest radiograph (figure 1). The final set of images for analysis consisted of 861 digital, posterior-anterior (PA) chest radiographs. Six of these radiographs were originally in a conventional film format and later digitized.

Group A (s+/c+ M.tb) and B (s−/c+ M.tb) included 194 (23%) of the study participants who were culture-positive for *Mycobacterium tuberculosis*. A further 233 patients (27%) presented with TB consistent symptoms but proved to be culture-negative with a sustained recovery after 5 months and therefore were classified as group F (Controls) (figure 1).

Overall, the prevalence of HIV was 44%. There was a significant difference between groups (p<0.01) with the highest prevalence (73%, 95%CI 58–84%) in group B (s−/c+ M.tb) and the lowest (34%, 95%CI 27–43%) in group A (s+/c+ M.tb). The proportion of patients who reported a prior history of TB was 17% overall, but differed significantly (p<0.01) between classifications and was highest (48%, 95%CI 28–68%) among group D (s−/c− clin.TB) (table 2).

**Table 2:** Summary statistics of study population.

	No data	All	Group A	Group B	Group C	Group D	Group E	Group F	Group G
			s+/c+ M.tb	s−/c+ M.tb	s±/c+ NTM	s−/c− clin.TB	EPTB	Controls	Indeterminate
**Characteristic**									
n (%)	0	861 (100)	146 (17)	48 (6)	134 (16)	25 (3)	5 (1)	233 (27)	270 (31)
Mean age (SD^1^)	0	42 (15)	37 (13)	41 (13)	42 (15)	46 (16)	37 (10)	42 (16)	44 (16)
Female sex n (%)	0	433 (50)	48 (33)	25 (52)	76 (57)	12 (48)	4 (80)	122 (52)	146 (54)
HIV positive n (%)	4	379 (44)	50 (34)	35 (73)	66 (49)	10 (40)	2 (40)	92 (39)	124 (46)
History of TB n (%)	0	144 (17)	17 (12)	6 (13)	27 (20)	12 (48)	0 (0)	26 (11)	56 (21)
**Symptoms at first visit**									
Cough ≥ 2 weeks n (%)	0	820 (95)	139 (95)	45 (94)	129 (96)	23 (92)	4 (80)	227 (97)	253 (94)
Night sweats n (%)	1	426 (50)	92 (63)	24 (50)	64 (48)	19 (79)	3 (60)	89 (38)	135 (50)
Haemoptysis n (%)	10	91 (11)	11 (8)	4 (8)	16 (12)	5 (20)	0 (0)	24 (10)	31 (12)
Fever n (%)	0	470 (55)	91 (62)	31 (65)	78 (58)	15 (60)	3 (60)	114 (49)	138 (51)
Weight loss n (%)	6	447 (52)	101 (70)	34 (71)	60 (45)	10 (40)	4 (80)	101 (44)	137 (51)

1 standard deviation.

Culture-positive individuals (group A (s+/c+ M.tb) + B (s−/c+ M.tb)) were significantly more likely to suffer from night sweats (60 vs. 38%), fever (63 vs. 49%) and weight loss (70 vs. 44%) than individuals classified as group F (Controls) (p<0.01). There was no evidence of a difference in the frequency of haemoptysis between these groups (p = 0.33).

**Figure 2:**
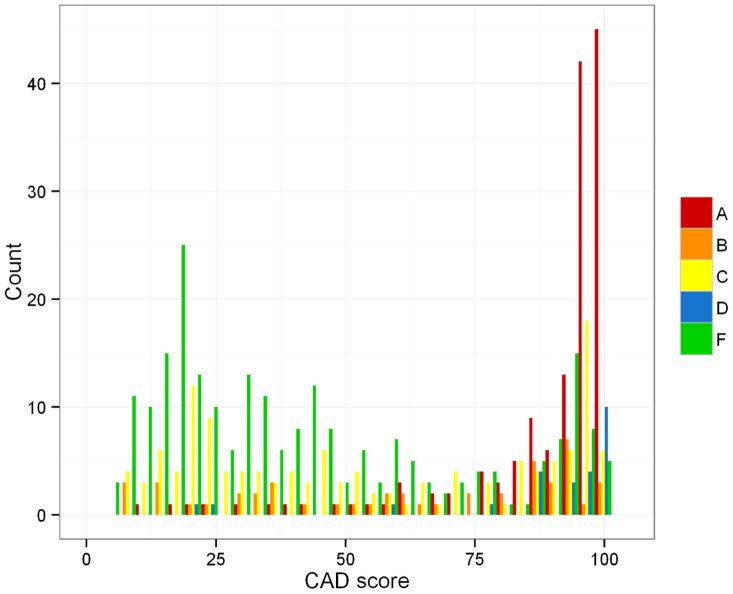
Distribution of CAD scores for patient groups A (s+/c+ M.tb), B (s−/c+ M.tb), C (s±/c+ NTM), D (s−/c− clin.TB) and F (Controls).

The distribution of CAD scores (figure 2) for group A (s+/c+ M.tb) and D (s−/c− clin.TB) tends towards higher scores, this is less marked for group B (s−/c+ M.tb). The scores attained by individuals classified as group C (s±/c+ NTM) and F (Controls) are clustered around lower values but can be found across the whole range. Around one third of the individuals of group F (Controls) did attain a CAD score greater than 50. On the whole there is considerable overlap in the distribution of CAD scores (table 3). The CAD scores in group B (s−/c+ M.tb) are significantly lower than those of group A (s+/c+ M.tb) and higher than those of group F (Controls) (p<0.01).

**Table 3:** Median CAD scores and 90% central range.

	**All**	**Group A**			**Group B**		
		**s+/c+ M.tb**			**s−/c+ M.tb**		
		**All**	HIV+	HIV-	**all**	HIV+	HIV-
Median CAD score	61	97	90	97	67	62	86
90% central range	11–100	44–100	33–100	60–100	11–99	13–98	12–96
	**Group C**	**Group D**	**Group E**	**Group F**	**Group G**		
	**s±/c+ NTM**	**s−/c− clin.TB**	**EPTB**	**Controls**	**Indeterminate**		
Median CAD score	48	97	83	34	57		
90% central range	13–98	32–100	38–92	9–95	11–100		

**Figure 3:**
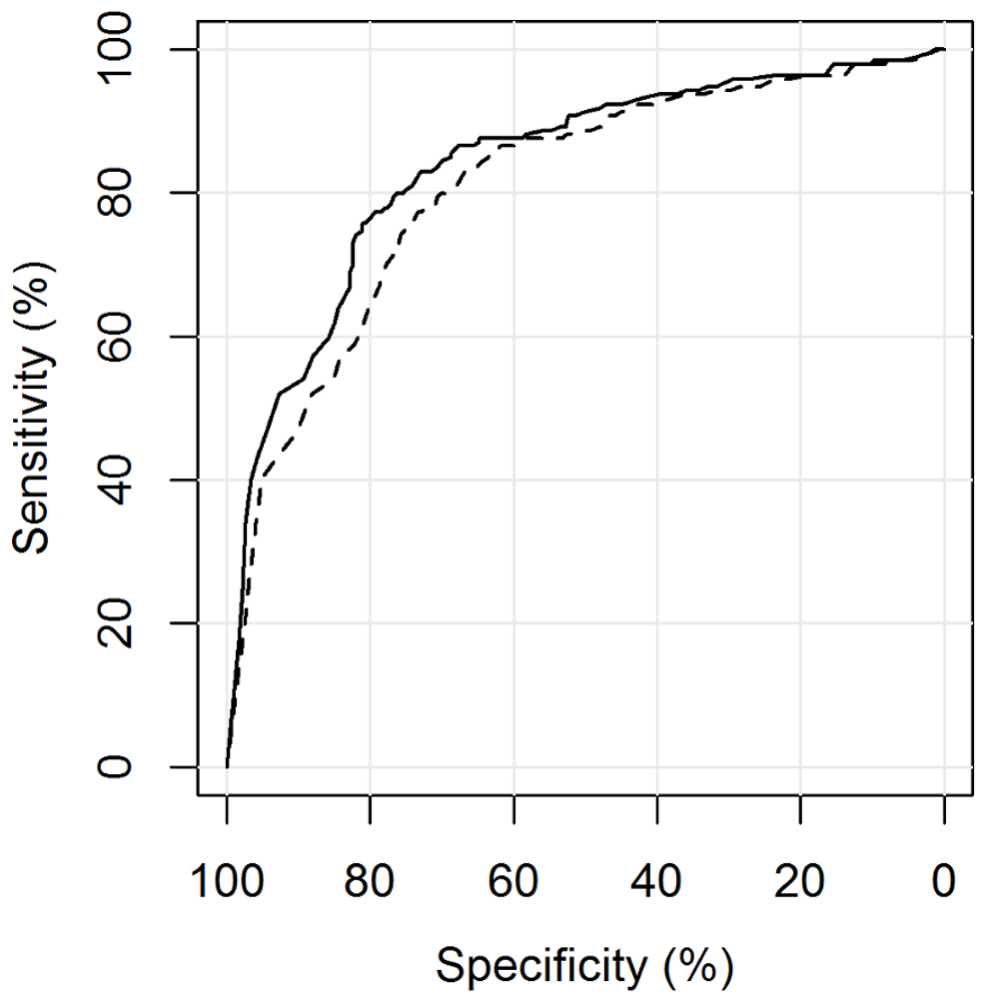
ROC analysis for the detection of M.tb culture-positive individuals. Legend. **—–**A (s+/c+ M.tb), B (s−/c+ M.tb) vs. F (Controls): A_z_ = 0.84 (0.80–0.88), **- - -** A (s+/c+ M.tb), B (s−/c+ M.tb) vs. C (s±/c+ NTM), E (EPTB), F (Controls): A_z_ = 0.81 (0.77–0.85), p = 0.28.

**Figure 4:**
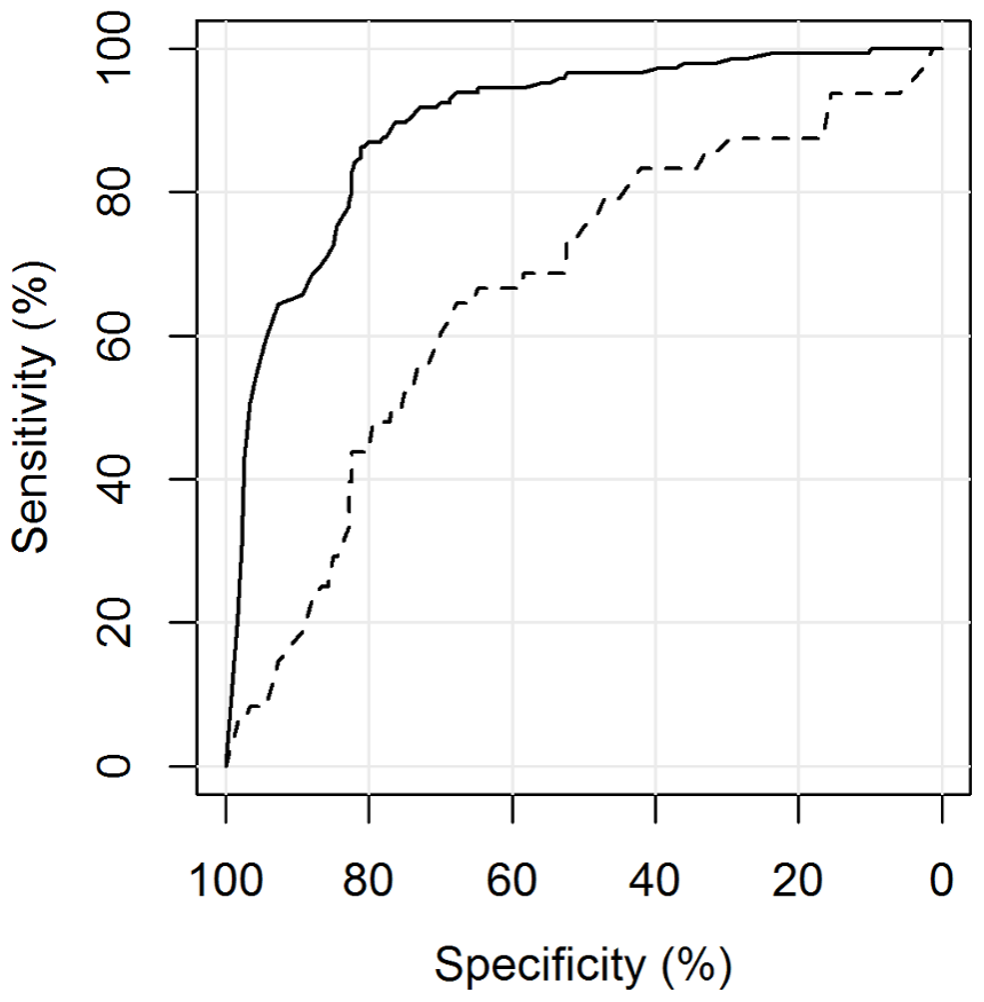
ROC analysis for the detection of M.tb culture-positive individuals by smear status. Legend. **—–** A (s+/c+ M.tb) vs. F (Controls): A_z_ = 0.90 (0.86–0.93), **- - -** B (s−/c+ M.tb) vs. F (Controls): A_z_ = 0.67 (0.58–0.75), p<0.01.

**Figure 5:**
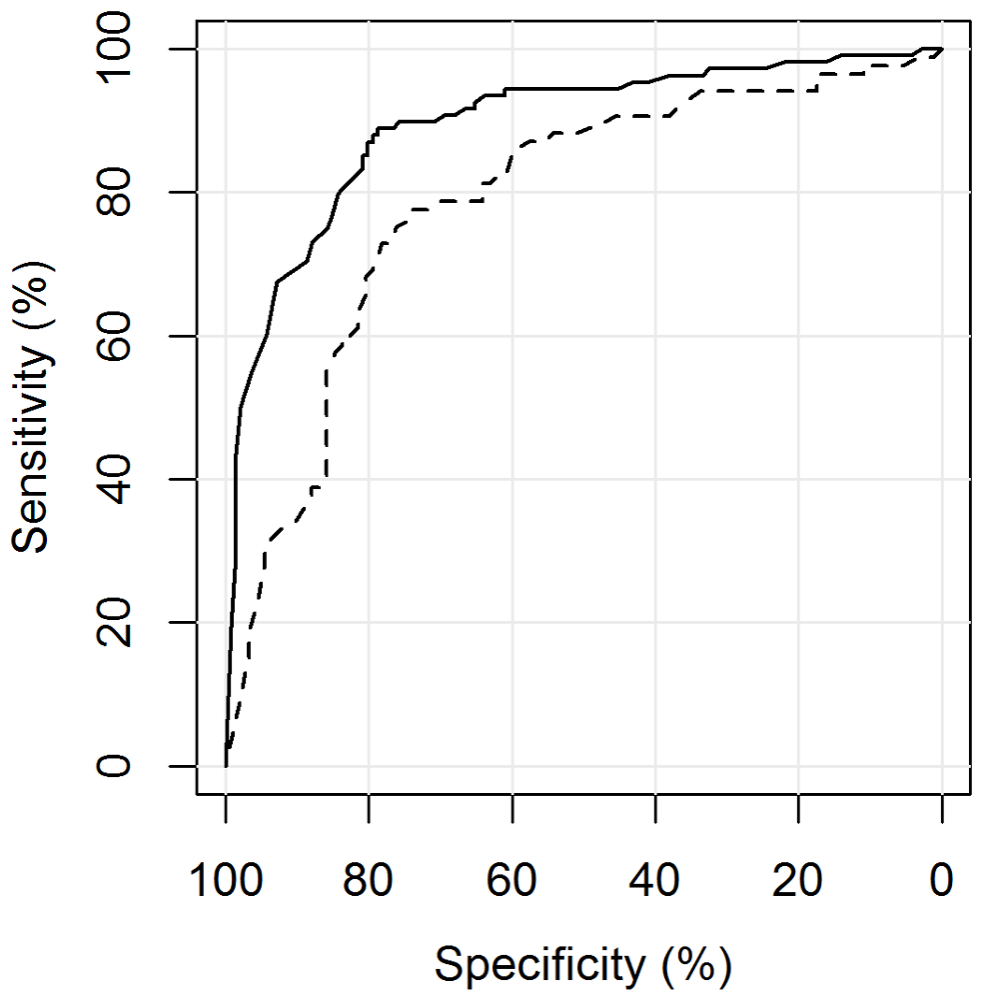
ROC analysis for the detection of M.tb culture-positive individuals by HIV Status. Legend. **—–** HIV negative. A (s+/c+ M.tb), B (s−/c+ M.tb) vs. F (Controls): A_z_ = 0.89 (0.85–0.94), **- - -** HIV positive. A (s+/c+ M.tb), B (s−/c+ M.tb) vs. F (Controls): A_z_ = 0.79 (0.72–0.86), p<0.01.

The automated reading software was able to distinguish between culture positive PTB cases (group A (s+/c+ M.tb) + B (s−/c+ M.tb)) and non TB patients (group F (Controls)) with an area under the curve of 0.84 (95%CI 0.80–0.88). Including all M.tb culture-negative patients (group C (s±/c+ NTM), E (EPTB) and F (Controls)) as the negative reference standard, CAD4TB performed slightly, but not significantly, worse: A_z_ = 0.81 (95%CI 0.77–0.85), p = 0.28 (figure 3). CAD4TB displayed a greater ability to differentiate smear-positive (group A (s+/c+ M.tb)) than smear-negative (group B (s−/c+ M.tb)) diseased individuals against non TB patients (group F (Controls)): A_z_ = 0.90 (95%CI 0.86–0.93) against A_z_ = 0.67 (95%CI 0.58–0.75), p<0.01 (figure 4). Similarly, the software distinguished diseased individuals (group A (s+/c+ M.tb) + B (s−/c+ M.tb)) from non TB patients (group F (Controls)) significantly more accurately among the HIV negative than among the HIV positive patient population: A_z_ = 0.89 (95%CI 0.85–0.94) against A_z_ = 0.79 (95%CI 0.72–0.86), p<0.01 (figure 5). Among group A (s+/c+ M.tb), B (s−/c+ M.tb) and F (Controls) there was no evidence of a difference in the performance of CAD4TB in between patients with and without history of TB: A_z_ = 0.84 (95%CI 0.80–0.89) against A_z_ = 0.79 (95%CI 0.65–0.92), p = 0.42. The area under the curve of CAD4TB for the discrimination of group B (s−/c+ M.tb) against C (s±/c+ NTM) was 0.56 (95%CI 0.46–0.65).

We calculated a set of cut-offs of the CAD4TB score for our patient population (table 4). For example, a cut-off of ≥74 leads to a sensitivity and specificity of CAD4TB of 77% (95%CI 71–83%) and 79% (95%CI 74–84%), respectively. Optimal values of sensitivity cannot be obtained without a considerable trade-off of specificity, and vice versa.

**Table 4:** Performance of CAD4TB and human readers.

	Threshold for test positivity	Sens.^1^ [%] (95%CI)	Spec.^2^ [%] (95%CI)	PPV^3^ [%] (95%CI)	NPV^4^ [%] (95%CI)	PLR^5^ (95%CI)	NLR^6^ (95%CI)
**CAD4TB**	≥23	95 (91–98)	33 (27–39)	54 (49–60)	89 (80–94)	1.42 (1.29–1.56)	0.16 (0.08–0.29)
	≥37	91 (86–94)	52 (46–59)	61 (55–67)	87 (80–92)	1.9 (1.65–2.19)	0.18 (0.11–0.28)
	≥56	85 (79–90)	69 (62–75)	69 (63–75)	85 (79–89)	2.71 (2.22–3.31)	0.22 (0.15–0.31)
	≥74	77 (71–83)	79 (74–84)	76 (69–82)	81 (75–86)	3.75 (2.88–4.88)	0.29 (0.22–0.37)
	≥89	62 (55–69)	85 (80–89)	77 (70–84)	73 (67–78)	4.12 (2.98–5.7)	0.45 (0.37–0.54)
	≥95	47 (40–54)	94 (91–97)	88 (80–93)	68 (63–73)	8.41 (4.86–14.56)	0.56 (0.49–0.64)
**Expert reader**	4	59 (52–66)	98 (95–99)	96 (91–99)	74 (69–79)	27.62 (11.52–66.26)	0.42 (0.35–0.49)
	3,4	78 (71–83)	85 (80–89)	81 (75–87)	82 (77–87)	5.18 (3.78–7.1)	0.26 (0.2–0.34)
	2,3,4	84 (78–89)	72 (65–77)	71 (65–77)	84 (79–89)	2.97 (2.4–3.67)	0.22 (0.16–0.31)
**Clinical officer**	4	7 (4–12)	97 (94–99)	70 (46–88)	56 (51–61)	2.8 (1.1–7.15)	0.95 (0.91–1)
	3,4	76 (69–82)	65 (58–71)	64 (58–70)	76 (70–82)	2.15 (1.78–2.61)	0.37 (0.29–0.49)
	2,3,4	97 (94–99)	18 (13–24)	50 (45–55)	89 (77–96)	1.19 (1.11–1.27)	0.14 (0.06–0.35)

1 sensitivity^ 2^specificity ^3^positive predictive value ^4^negative predictive value ^5^positive likelihood ratio ^6^negative likelihood ratio. All parameters were assessed against group A and B as positive reference standard and group F as negative controls.

**Figure 6:**
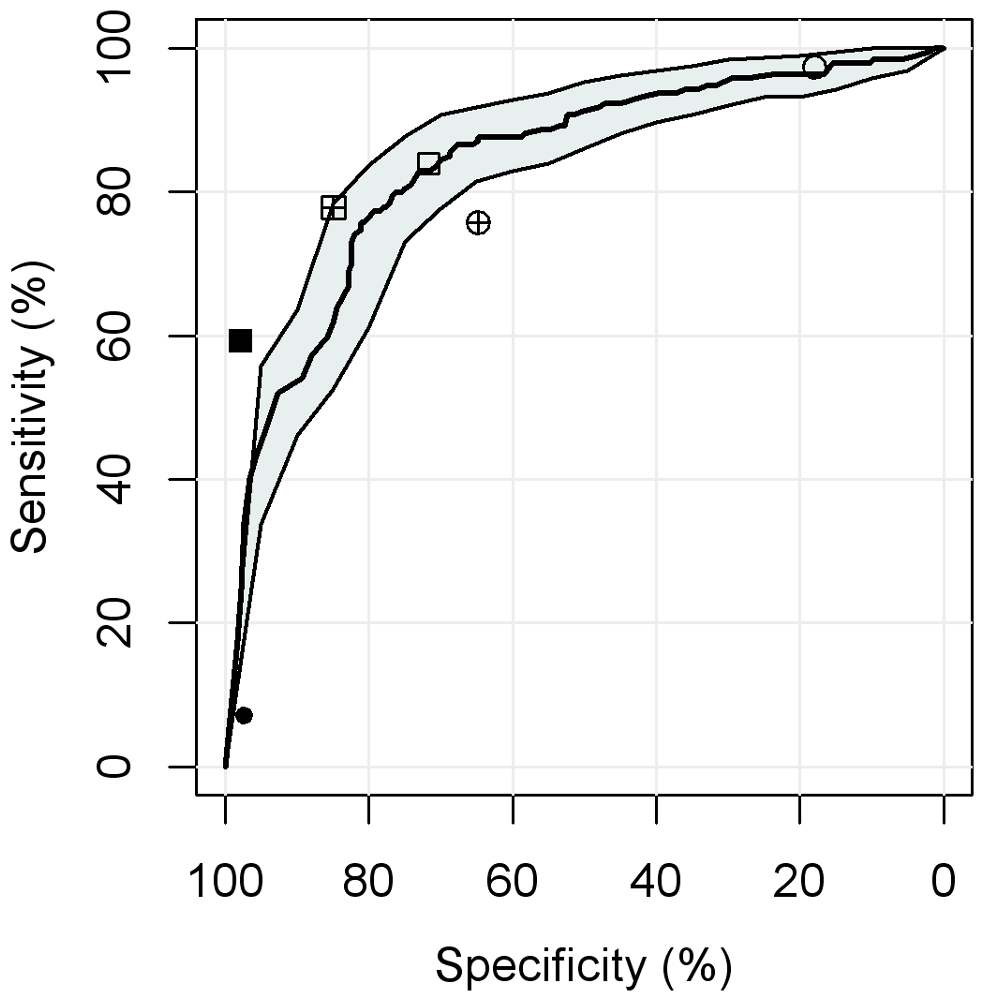
Comparison of automated and human reading. Legend. Sensitivity and specificity to distinguish group A (s+/c+ M.tb) and B (s−/c+ M.tb) vs. F (Controls). Line and shaded area: ROC curve and 95% CI for CAD4TB. The expert reader is represented by square symbols, the clinical officer by round symbols. The different fill of the symbols indicate different reading thresholds: empty symbols  = ‘any abnormality’, crossed symbols  = ‘TB consistent abnormalities’ and filled symbols  = ‘abnormalities highly suggestive for TB’.

Setting the CAD4TB cut-off to give sensitivity values achieved by human readers allowed us to compare the performance of automated and human readings (figure 6). There was no evidence of a difference between the specificities achieved by the software and both human readers reporting ‘any abnormality’ (p = 0.49, 0.88). This was different for tuberculosis specific reporting thresholds: CAD4TB was significantly more specific (p = 0.02) than the clinical officer reporting ‘TB consistent abnormalities’ but did not reach the accuracy level of the expert reader (p = 0.02).

A review, carried out by a third reader (senior radiologist with extensive experience in TB), of the images (n = 7) rated as false negative by CAD4TB at the exemplary cut-off (<74) but as true positive (conclusion 3+4) by both human readers did not reveal any obvious pattern of abnormalities missed by CAD4TB.

## Discussion

Automating the interpretation of a chest radiograph for the detection of active pulmonary tuberculosis leads to objective, reproducible results and a standardized way of reporting. The main finding of our study is that the automated reading software CAD4TB (v3.07, updated release) achieved a good diagnostic accuracy (A_z_ = 0.84 (95%CI 0.80–0.88)) on a large set of CXRs of presumptive TB patients from sub-Saharan Africa. The accuracy of CAD4TB was slightly, but not significantly, worse in our secondary analysis using a binary classification of patients (M.tb culture-positive vs. negative) which we included for a better comparability with other diagnostic accuracy studies.

In our study, performance of automated and human reading was comparable when the observers considered ‘any abnormality’. For a more TB specific reading threshold, however, the software outperformed the clinical officer significantly but did not reach the accuracy of the expert reader. The software identified a significantly higher proportion of smear-positive compared to smear-negative, culture-positive individuals - most likely because smear-negative PTB patients tend to have more discrete or atypical radiographic features, especially in combination with HIV infection [25]. This assumption is substantiated by the fact that CAD4TB detected PTB cases significantly more accurately among HIV negative than HIV positive individuals.

Identifying cases of active PTB among symptomatic individuals with abnormal CXRs due to other pulmonary conditions (e.g. pneumonia) or sequelae of tuberculosis remains challenging for both human and automated readers. This fact manifests itself in low specificity values as a consequence of the considerable overlap in the distribution of CAD scores for the defined groups and the far higher proportion of patients who reported a history of TB among group D (s−/c− clin.TB).

One of the strengths of our study is the direct comparison of automated and human reading on the same set of images. Due to inter-reader variability in the interpretation of chest radiographs and the ability to include only one clinical officer and expert reader, the degree to which this comparison can be generalized is strongly limited. It is possible that other clinical officers or expert readers would have outperformed the software in our study. A second limitation of our study is the fact that it was conducted in only one high burden country and it would be preferable to repeat it in different settings to assess generalizability of the results.

HIV infection seems to influence the diagnostic accuracy of CAD4TB, so our findings cannot be readily generalized to populations that differ significantly in their HIV prevalence. A further constraint of the study is the high proportion (31%, group G) of patients who either could not be followed up sufficiently to comply with the precise classification criteria or that were still non TB patients but symptomatic after five months and therefore could not be classified as group F (Controls). However, since a heterogeneous patient group is concerned and the data can be most likely assumed to be missing at random, it can be postulated that study results were not substantially influenced.

The relatively high number of patients that were found to be culture-positive for NTMs (16%, group C) is not uncommon in the sub-Saharan African context [26]–[28]. This is probably largely due to contamination of culture samples either at patient level or from the environment as only few patients suffered from a pathogenetic relevant NTM infection that fulfilled the diagnostic criteria for a Nontuberculous Mycobacterial Lung Disease according to the American Thoracic Society [29]. The inability of CAD4TB to differentiate between patients of group B (s−/c+ M.tb) and C (s±/c+ NTM) might be due to the heterogeneity of group C (s±/c+ NTM).

Maduskar et al. evaluated the performance of a previous CAD4TB version and compared it to both, clinical officers rating the radiograph between 0–100 and the binary decision of an expert reader (as radiological reference) for the presence of TB consistent abnormalities [11]. We decided to use hierarchical reading thresholds as we believe that this reflects the common radiological practice in a setting like ours. The high accuracy achieved for the radiological reference (A_z_ = 0.91 (95%CI 0.86–0.95)) [11] is consistent with our finding that CAD4TB approaches values of sensitivity and specificity achieved by the expert reader. The diagnostic accuracy of CAD4TB for the bacteriological reference was higher using the newer version in our study compared to previous CAD4TB versions used in the study of Maduskar and another small scale study [29]. This suggests advancement in the development of the software, which is especially encouraging as we evaluated its performance on images obtained from a different X-ray machine than the one it was originally developed for.

Current national diagnostic algorithms for presumptive adult TB patients in many sub-Saharan African countries request between two to six negative sputum smear examinations and a failed treatment with a broad-spectrum antibiotic for 7 days before a chest X-ray is ordered [31]–[38]. According to recommendations of the World Health Organization (WHO), the CXR exam should even precede an administration of antibiotics in settings where HIV is highly prevalent and resources are constrained [39]. In both cases a thorough X-ray report and its integration with clinical information by a medically trained person is needed for the final diagnosis of smear-negative PTB. Our findings indicate that in this situation the CAD4TB software could assist less experienced readers in their judgment. It could not entirely replace the human interpretation for radiographic questions beyond that of active tuberculosis as the software was not designed to detect other pathologies. Its output, a single number, does not reflect the presence of abnormalities unrelated to TB, whose detection might be not less important or even prompt immediate action (such as pneumothorax). In addition, the high proportion of patients of group F (Controls) who did attain a ‘false positive’ high CAD score due to other pulmonary pathologies as pneumonia have to be taken into consideration.

By contrast, the very condensed output of the automated reading might be preferable for the binary decision in screening situations of either conveying a screened individual to confirmatory testing or to declare the absence of PTB. A strong feature of CAD4TB in its current stage of development is its continuous output, which allows adjusting the reading threshold to the purpose of use, local epidemiology and availability of resources (such as the capacity to perform smear microscopy or the number of Xpert MTB/Rif cartridges).

It is not known whether active screening will have a positive effect on TB epidemiology [40]; however, the slow decline in incidence and case detection gap suggest that a more active approach could complement patient-initiated pathways [41] and enhance their efficacy. Among the broad spectrum of possible active case finding strategies, the new comprehensive WHO guidelines for the systematic screening for active tuberculosis among certain risk groups, in which chest radiography found its firm place, if available, as a first or second screening step [42]. A robust CAD has the potential to enhance and facilitate the implementation of these recommendations by ensuring high test standards of objectivity, reproducibility and accuracy without straining personnel resources. A prerequisite for the CAD application is the availability of digital radiography, which is not yet the case in most of resource-constrained high-burden settings. However, it has been identified as a key action point in a WHO Workshop to Scale Up the Implementation of Collaborative TB/HIV Activities in Africa earlier this year [43] as it has been shown to be feasible and to result in a significant better quality of chest radiography compared to conventional X-ray technology in countries with limited resources [44].

Prospective studies on cost-effectiveness, operational and ethical aspects of the use of CAD in different high burden countries are needed. Future research should also address the question whether the integration of a CAD output with clinical variables like symptoms and risk factors could result in a more accurate screening step.

In conclusion, the computer-aided diagnosis system CAD4TB is a reproducible and accurate test for the detection of pulmonary tuberculosis on radiographs in symptomatic patients. This prompts additional research on how its potential, both as assistance for clinical officers in the diagnostic interpretation of radiographs and as standalone triage test in systematic screening settings, can be exploited.
